# Dynamically induced friction reduction in micro-structured interfaces

**DOI:** 10.1038/s41598-021-87484-5

**Published:** 2021-04-14

**Authors:** N. Menga, F. Bottiglione, G. Carbone

**Affiliations:** 1 Department of Mechanics, Mathematics and Management, Politecnico of Bari, V.le Japigia, 182, 70126 Bari, Italy; 2 Department of Mechanical Engineering, Imperial College London, Exhibition Road, London, SW7 2AZ UK; 3 Physics Department “M. Merlin”, CNR - Institute for Photonics and Nanotechnologies U.O.S. Bari, via Amendola 173, 70126 Bari, Italy

**Keywords:** Mechanical engineering, Nanoscale devices, Applied physics

## Abstract

We investigate the dynamic behavior of a regular array of *in-plane* elastic supports interposed between a sliding rigid body and a rigid substrate. Each support is modelled as a mass connected to a fixed pivot by means of radial and tangential elastic elements. Frictional interactions are considered at the interface between the supports and the sliding body. Depending on the specific elastic properties of the supports, different dynamic regimes can be achieved, which, in turn, affect the system frictional behavior. Specifically, due to transverse microscopic vibration of the supports, a lower friction force opposing the macroscopic motion of the rigid body can be achieved compared to the case where no supports are present and rubbing occurs with the substrate. Furthermore, we found that the supports static orientation plays a key role in determining the frictional interactions, thus offering the chance to specifically design the array aiming at controlling the resulting interfacial friction force.

## Introduction

Controlling the tribological behavior of interfaces has been one of the major concerns in modern engineering. This is because, friction is always a primary source of energy dissipation in industrial processes, thus resulting in high energy loss and reduced cost-efficiency. However, since the tribological properties of the contacting surfaces are governed by complex phenomena occurring at the interface which, in turns, depend on several parameters (e.g. roughness, hardness, contact configuration, lubricating condition, contacting material pair, etc.), during the last decades, several research paths have been explored aiming at controlling the resulting interfacial friction. It is the case, for instance, of the numerous investigations involving viscoelastic energy dissipation and friction, which have seen an always increasing accuracy in predicting the frictional behavior of rubber-like interfaces^1–5^. Similarly, also the case of lubricated contacts has been deeply investigated, inferring that surface micro-texturing is probably one of the most promising methods to reduce lubricated friction^6–9^. Interestingly, in the case of soft wet interfaces, the effect of surface micro-texturing is to alter the elastohydrodynamic regime of the contact. Consequently, at low speed and low normal load, a significant friction reduction can be achieved, compared to the case of smooth interfaces^10,11^; on the contrary, at high speed, increased friction has been reported.

Surface texturing has also been increasingly utilized to control the tribological behavior of dry interfaces. This mostly refers to the possibility to control the behavior of the contact interface by developing meta-materials with specific local material properties, such as high interfacial compliance. Such an interface specialization can be pursued, for instance, by micro-structuring the surfaces in micro-pillars and nanofibers with specific aspect ratio, size, and orientation. Recent studies^12–14^ have experimentally investigated the effect of the interfacial micropillars geometry on dry friction between both soft and hard contact pairs (namely steel and low-density polyethylene), showing that specific design conditions are able to produce an effective friction reduction. Similarly, experimental measurements have also been performed in smaller structures^15,16^, such as polymeric nano hairs and nanofibers opportunely machined via colloidal lithography, aiming at highlighting the effect of the micro-structures aspect ratio on the frictional behavior. Also, the effect of areal density of microstructures on the contact interface has been investigated with an application, for instance, to tongue/palate tribological behavior^17^, showing that dry friction significantly decreases with the density increasing. Notably, similar results have also been reported in the case of dry contacts involving hard interfaces^18^. Moreover, building on the same path, further investigations^19^ have focused on tuning the micropillar tapering to control both adhesion and friction in bio-inspired adhesive strips, showing that also the effective contact area reduction achieved in highly tapered structure contributes to the reported friction reduction. Friction reduction has also been achieved in relatively soft elastomeric contacts by means of surface texturing with a hexagonal pattern^20^ which induces a significant reduction of the real contact area via micro-structures bending, as reported also in Ref.^21^.

Interestingly, only recent studies have focused on possible dynamic effects on friction reduction in micro-/nano-textured interfaces. Indeed, it is well known that, even in the exemplar case of rough interfaces, vibrations can significantly reduce interfacial friction in sliding contact mechanics^22,23^. This can be ascribed to the emergence of non-negligible reciprocating microscopic motion superimposed to the macroscopic sliding which, depending on the relative angle, may lead to a non-vanishing transverse component of the friction force which results in a reduction of the friction component opposing the macroscopic sliding^24,25^. Further studies on the same topic have shown that the interface stiffness plays a key role in determining the amount of friction reduction achievable when transverse vibration is superimposed to sliding^26^. Similarly, in the assumption of microscopic constant Coulomb friction and normal oscillations, it has been shown that a characteristic velocity can be found above which the oscillations do not affect the macroscopic friction coefficient^27^. In this regard, micro-structured surfaces offer the opportunity to tailor the stiffness and the dynamic properties of the interface to achieve a stronger friction control. Indeed, dynamic models based on lumped-elements description of the micro-structured interface can be used to opportunely tune the microstructure geometry. It is the case, for instance, of Ref.^28^, where sliding friction measurements on soft micropillars have been compared to a simple dynamic model, showing a good result agreement. Similarly, in a series of paper^29, 30^, the dynamic effects of hierarchical and anisotropic interfacial microstructures on 2-D problems have been numerically investigated by relying on mass-spring models, showing that the resulting friction strongly depends on the size, shape and orientation of the micro-structures.

**Figure 1:**
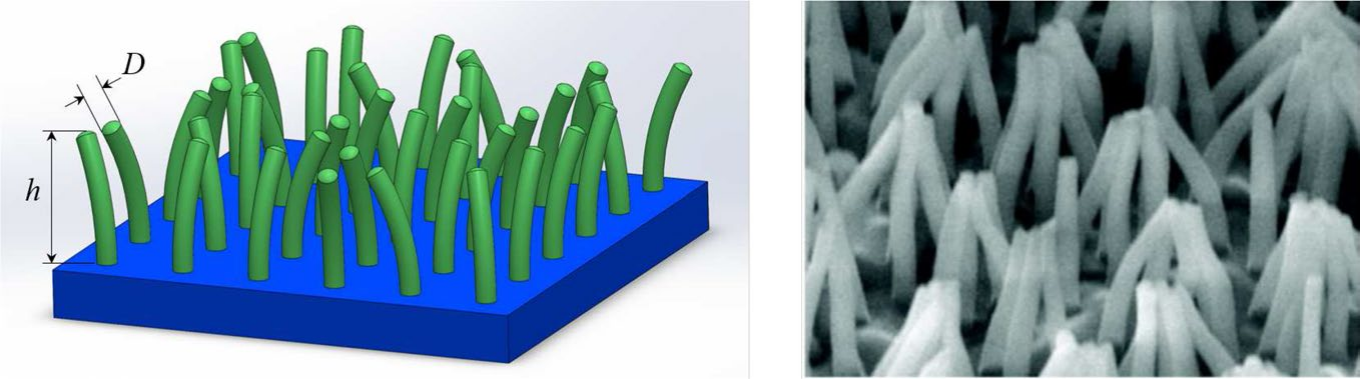
Left side: an example of micro-structured surface with bristle-like micropillars. Right side: a SEM image of ordered low-density polyethylene (LDPE) nanofiber arrays adapted with permission from Ref.^12^. Copyright 2011 American Chemical Society.

In this view, building on several investigations dealing with bristle-like^31^ and mushrooms-like^32^ interfacial microstructures, here we focus on a micro-structured 2D surface made of curved micropillars with random orientation, such as that shown in Fig. 1. The micropillars *in-plane *elastic behavior can be modelled by means of the combination of radial and torsional elastic elements, whose equivalent stiffness can be calculated in the framework of linear elastic beam theory as $k_{r}\approx ED^{4}/h^{3}$ and $k_{\theta }\approx GD^{4}/h$ (with *D* and *h* being the micropillar diameter and height, and *E* and *G* being the Young and shear elastic moduli). The system also consists of a rigid block sliding on the bristle array in the presence of interfacial friction. The resulting model provides insight into the *in-plane* dynamic response of the micro-structured interface during sliding, thus allowing to predict the effect of the dynamic parameters on the resulting *in-plane* tangential force opposing the macroscopic sliding of the rigid block.

## Formulation

**Figure 2:**
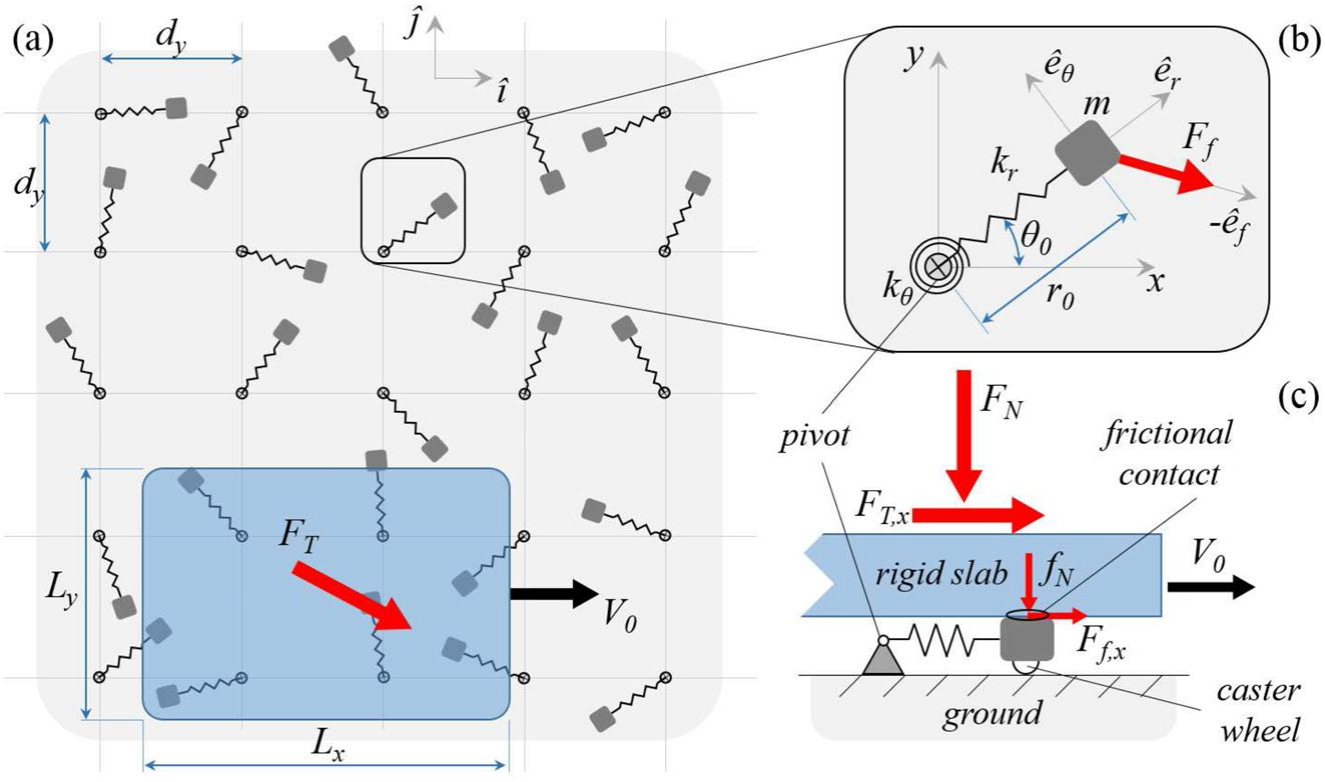
The functional scheme of the system at hand: a rigid sliding slab is borne by an array of *in-plane* elastic supports. (**a**) is a top view of the whole system; whereas (**b**) is a close-up to a generic support of mass *m*, radial and torsional stiffness $k_{r}$ and $k_{\theta }$, static length $r_{0}$ and static orientation $\theta _{0}$. (**c**) is a side view of the contact between the slab and a generic support. Notably, $F_{T,x}={\mathbf {F}}_{T}\cdot {\hat{{\imath }}}$, and $F_{f,x} ={\mathbf {F}}_{f}\cdot {\hat{{\imath }}}$.

In Fig. 2a we show the functional scheme of the system under investigation. A rigid slab is sliding at a velocity $V_{0}$ in the *x-direction* under the action of a normal load ${\mathbf {F}}_{N}$ and an *in-plane *driving force ${\mathbf {F}}_{T}$. The slab is borne, under dry conditions, by an array of elastic supports with spacing length $d_{x}$ and $d_{y}$. Each support is composed of a mass *m* connected to a pivot by a radial spring (with stiffness $k_{r}$ and equilibrium length $r_{0}$) and a torsion spring (with stiffness $k_{\theta }$ and equilibrium orientation $\theta _{0}$). Moreover, the distribution of the supports’ static orientations $\theta _{0}$ is homogeneous within the array.

Since in this study we are interested in investigating the *in-plane* dynamics of the bristle-like elements (see Fig. 1) whose tips do not directly contact with the underneath substrate, we consider pure normal interactions between the supports and the underlying ground (i.e. frictionless conditions), as those achievable by interposing castor wheels. On the contrary, frictional interactions occur at the interface between the elastic supports and the slab (see Fig. 2c). Furthermore, we neglect any interaction between the supports.

### The dynamics of the elastic supports

With reference to Fig. 2b, the *in-plane* momentum balance of a generic elastic support gives

1 $$\begin{aligned} m\ddot{\mathbf {x}}=-\left[ k_{r}(r-r_{0})-\phi \left( r\right) \right] {\hat{\mathbf {e}}}_{r}-\frac{k_{\theta }}{r}(\theta -\theta _{0}){\hat{\mathbf {e}} }_{\theta }+{\mathbf {F}}_{f}, \end{aligned}$$

where ${\mathbf {x}}\left( t\right) =x{\hat{{\imath }} }+y{\hat{{\jmath }}}$ is the mass position vector with ${\hat{{\imath }}}$ and ${\hat{{\jmath }}}$ unit vectors along *x-* and *y-axis* respectively (see Fig. 2a), ${\mathbf {F}}_{f} =F_{f}{\hat{\mathbf {e}}}_{f}$ is the friction force acting on the support, $r=\left| {\mathbf {x}}\right| =\sqrt{x^{2}+y^{2}}$, $\theta$ is the support dynamic orientation, and

2 $$\begin{aligned} {\hat{\mathbf {e}}}_{r}&=\left( \frac{x}{r},\frac{y}{r}\right) ,\nonumber \\ {\hat{\mathbf {e}}}_{\theta }&=\left( -\frac{y}{r},\frac{x}{r}\right) ,\nonumber \\ {\hat{\mathbf {e}}}_{f}&=\frac{{\mathbf {v}}_{R}}{\left| {\mathbf {v}} _{R}\right| }, \end{aligned}$$

with ${\mathbf {v}}_{R}= \dot{\mathbf {{x}}}-V_{0}{\hat{\mathbf {\imath }}}$ being the relative velocity between the elastic support and the rigid slab. Moreover, in Eq. (1) the function

3 $$\begin{aligned} \phi \left( r\right) =\frac{\alpha }{2}\frac{mV_{0}^{2}}{\lambda } e^{-r/\lambda } \end{aligned}$$

represents a short-range repulsive term which, by opportunely tuning the range parameter $\lambda$, simulates the physical requirement of a non-vanishing radial spring length, also avoiding the singular behavior of Eq. (1) in ${\mathbf {x}}=\left( 0,0\right)$. Furthermore, a sensitivity analysis on the effect of the parameter $\alpha$ on the numerical stability of the solution has been performed, eventually indicating that $\alpha =1$ is well-suited for the case at hand. Regarding the normal load $f_{N}$ acting on each support, here we assume the total normal load $F_{N}$ acting on the slab to uniformly distribute over the effective number $N_{c}$ of supports in contact with the slab, therefore $f_{N}=F_{N}/N_{c}$. Finally, the modulus of the friction force acting on a single support is given by $F_{f}=-f_{N}\mu \left( v_{R}\right)$, with $\mu \left( v_{R}=\left| {\mathbf {v}}_{R}\right| \right)$ being the friction coefficient.

**Figure 3:**
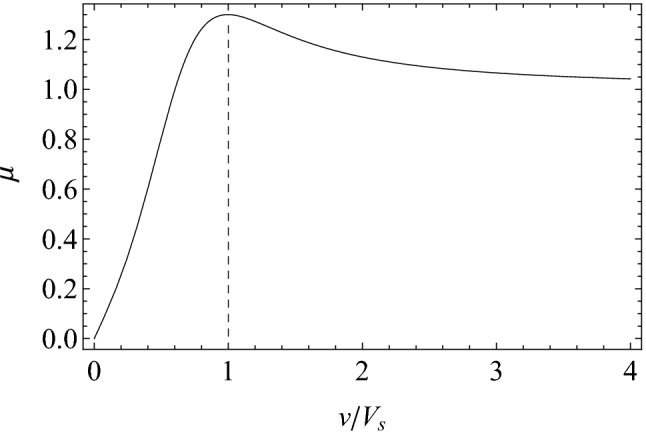
The friction coefficient as a function of the ratio $v/V_{s}$, with $V_{s}$ being the static velocity tolerance. Parameters are $\mu _{s}=1.3$, $A=1.44$, $B=-20$.

Since we expect that the supports dynamics leads to non-steady frictional interactions during the slab sliding, we adopt a velocity-based friction model whose non-monotonic trend, in agreement with Refs.^33,34^, represents a good approximation of the real rubber-like materials interfacial frictional behavior^35^. The friction coefficient is shown in Fig. 3 and is given by

4 $$\begin{aligned} \mu \left( v_{R}\right) =\mu _{s}\sin \left( A\tan ^{-1}\left\{ \zeta v_{R}-B\left[ \zeta v_{R}-\tan ^{-1}(\zeta v_{R})\right] \right\} \right) , \end{aligned}$$

where *A*, *B* and $\zeta$ are empirical coefficients, and $\mu _{s}$ represent the “static” friction coefficient occurring at the velocity $V_{s}=1/\zeta$.

#### Dimensionless quantities

For the sake of simplicity, it is convenient to rewrite Eqs. (1) – (3) in a dimensionless form, so the following dimensionless parameters are adopted: ${\tilde{\mathbf {x}}=x}/r_{0}$, ${\tilde{r}}=r/r_{0}$, ${\tilde{\lambda }}=\lambda /r_{0}$, ${\tilde{k}}_{r}=r_{0}^{2}k_{r}/\left( mV_{0} ^{2}\right)$, ${\tilde{k}}_{\theta }=k_{\theta }/\left( mV_{0}^{2}\right)$, and ${\tilde{F}}_{f}=r_{0}F_{f}/\left( mV_{0}^{2}\right)$. The dimensionless time is $\tau =tV_{0}/r_{0}$, so that the dimensionless velocity and acceleration take the forms ${\tilde{\mathbf {v}}}=d{\tilde{\mathbf {x}}/} d\tau ={\dot{\mathbf {{x}}}}/V_{0}$ and ${\tilde{\mathbf {a}}}=d{\tilde{\mathbf {v}} /}d\tau =\ddot{\mathbf {x}}r_{0}/V_{0}^{2}$, respectively. Finally, we have

5 $$\begin{aligned} {\tilde{\mathbf {a}}}=-\left[ {\tilde{k}}_{r}({\tilde{r}}-1)-{\tilde{\phi }}\left( {\tilde{r}}\right) \right] {\hat{\mathbf {e}}}_{r}-\frac{{\tilde{k}}_{\theta } }{{\tilde{r}}}(\theta -\theta _{0}){\hat{\mathbf {e}}}_{\theta }+{\tilde{F}} _{f}{\hat{\mathbf {e}}}_{f}, \end{aligned}$$

where ${\tilde{\phi }}\left( {\tilde{r}}\right) =\left( 2{\tilde{\lambda }}\right) ^{-1}e^{-{\tilde{r}}/{\tilde{\lambda }}}$. Moreover, we define ${\tilde{F}}_{N} =r_{0}F_{N}/\left( N_{c,0}mV_{0}^{2}\right)$, so that ${\tilde{F}}_{f} =-\beta {\tilde{F}}_{N}\mu \left( v_{R}\right)$ with $\beta =N_{c,0}/N_{c}$.

Equation (5) represents a set of nonlinear differential equations describing the *in-plane* motion of each support, however an addition equation in $\theta \left( t\right)$ is needed to mathematically define the problem. This is provided in term of a linear first-order ODE as

6 $$\begin{aligned} \frac{d\theta }{d\tau }=\frac{{\tilde{x}}{\tilde{v}}_{y}-{\tilde{y}}{\tilde{v}}_{x} }{{\tilde{r}}^{2}}. \end{aligned}$$

### The slab main quantities

**Figure 4:**
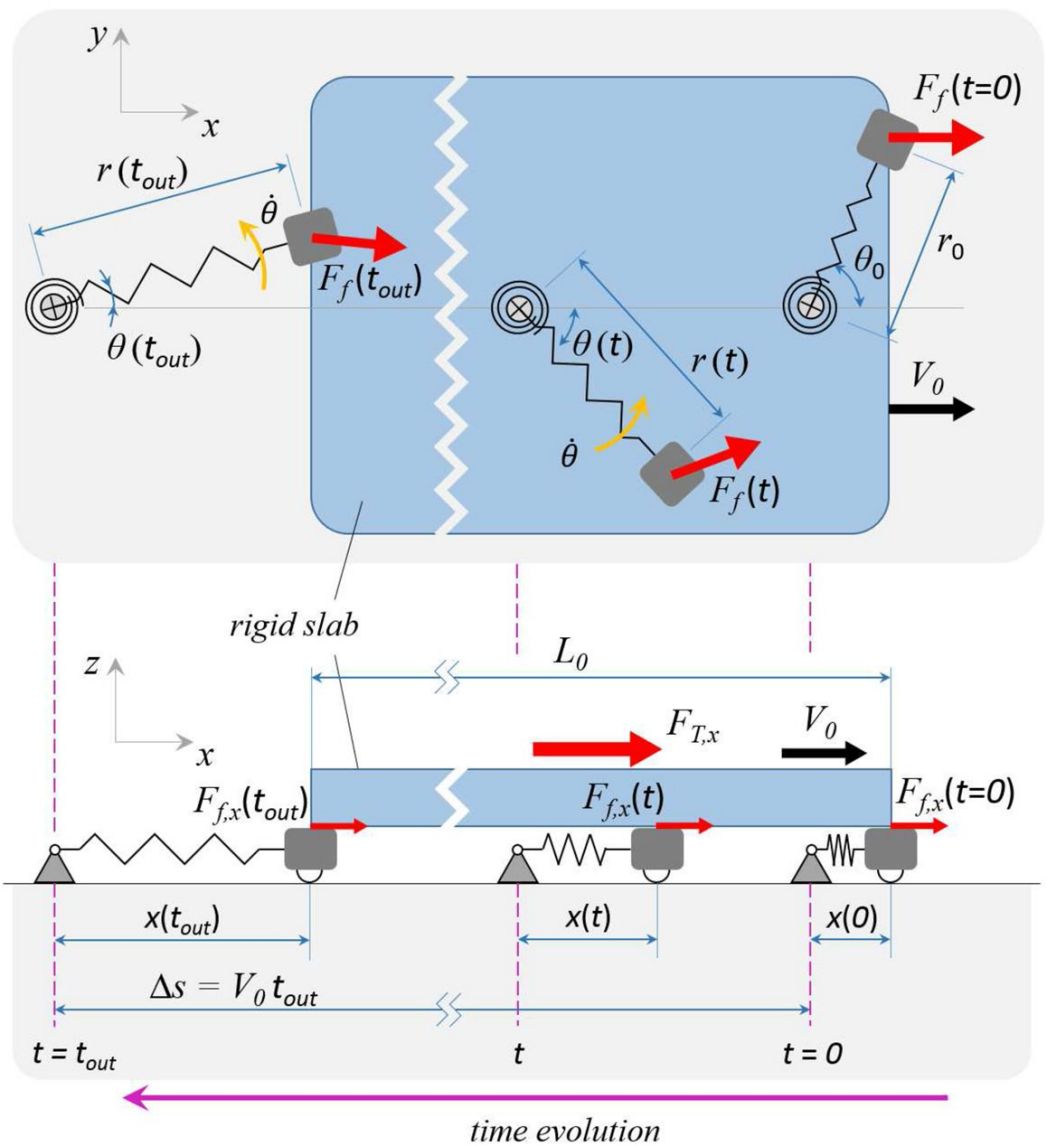
A scheme of the time evolution of the relative sliding between the rigid slab and a single elastic support. Contact starts at time $t=0$ and ends at time $t=t_{out}$. Moreover, $F_{T}$ is the constant driving force acting on the slab, whereas $F_{f}\left( t\right)$ is the istantaneous friction force experienced by the support. Top view shows the angular oscillation and radial elongation of the supports, and side view allows to appreciate the supports longitudinal dynamics.

The *in-plane* dynamics of each elastic support is defined through Eqs. (5) – (6). In this section, we focus on the main forces acting on the rigid slab due to the relative motion, and the resulting friction, occurring between the latter and the elastic supports, as indeed shown in Fig. 4.

In order to calculate these forces, we should consider the effect of each single support in contact underneath the slab, with its own dynamic properties, location and local sliding condition. However, all the supports share the same elastic and frictional properties, and the distribution of the static orientations $\theta _{0}$ is homogeneous within the array. Consequently, since we are interested in the steady sliding response, we can simplify the problem considering, as representative of the whole array frictional behavior, the average friction arising from the dynamic evolutions of a sufficiently large number $n\gg N_{c,0}$ (notably, $N_{c,0}=L_{x} L_{y}/d_{x}d_{y}$) of supports each of which is calculated from the time ($t=0$) at which the support is approached by the slab to when ($t=t_{out}$) it is released from the contact. Therefore, according to Fig. 4, under steady sliding conditions, the *in-plane* rigid slab equilibrium gives

7 $$\begin{aligned} {\mathbf {F}}_{T}=-\sum _{\eta =1}^{n}\left( {\bar{\mathbf {F}}}_{f}\right) _{\eta }, \end{aligned}$$

where $\left( {\bar{\mathbf {F}}}_{f}\right) _{\eta }$ represents the mean friction force associated with the $\eta$-th support in contact with the slab so that, per each value of $\eta =1,2,\ldots ,n$, we have

8 $$\begin{aligned} {\bar{\mathbf {F}}}_{f}=\frac{1}{t_{out}}\int _{0}^{t_{out}}{\mathbf {F}}_{f}\left( t^{\prime }\right) dt^{\prime }. \end{aligned}$$

The value of $t_{out}$ of each contact can be numerically calculated as

9 $$\begin{aligned} L_{0}=-\int _{0}^{t_{out}}{\mathbf {v}}_{R}\cdot {\hat{\mathbf {\imath }}.} \end{aligned}$$

The *in-plane* averaged normalized friction force components acting on the slab can then be calculated as ensemble average among the *n* considered supports as

10 $$\begin{aligned} \frac{F_{T,x}}{F_{N}}&=\left\langle \frac{{\bar{\mathbf {F}}}_{f} \cdot {\hat{\mathbf {\imath }}}}{f_{N}}\right\rangle , \end{aligned}$$

11 $$\begin{aligned} \frac{F_{T,y}}{F_{N}}&=\left\langle \frac{{\bar{\mathbf {F}}}_{f} \cdot {\hat{{\jmath }}}}{f_{N}}\right\rangle , \end{aligned}$$

where $$F_{T,x}={\mathbf {F}}_{T,x}\cdot {\hat{\mathbf {\imath }}}$$, and $$F_{T,y} ={\mathbf {F}}_{T}\cdot {\hat{{\jmath }}}$$

It is worth observing that the resulting value of $F_{T,x}/F_{N}$ and $F_{T,y}/F_{N}$ still slightly oscillate even under steady sliding conditions. This mainly depends on the fact that the new supports getting into contact with the slab as this advances have a static orientation $\theta _{0}$, resulting in different dynamic behavior. However, in the limit of sufficiently large values of *n*, the oscillation asymptotically vanishes.

Moreover, due to the frictional interaction between the slab and the elastic supports, a significant part of the work per unit time ${\dot{W}}_{s}$ done by the force driving ${\mathbf {F}}_{T}$ is dissipated. Indeed, the energy balance per unit time of the whole system gives

12 $$\begin{aligned} {\dot{W}}_{s}=F_{T,x}V_{0}={\dot{D}}_{x}+{\dot{D}}_{y}+{\dot{U}}_{el}+{\dot{U}}_{k}, \end{aligned}$$

where

13 $$\begin{aligned} {\dot{D}}_{x}&=-\left\langle \int _{0}^{t_{out}}\left[ {\mathbf {F}}_{f}\left( t^{\prime }\right) \cdot {\hat{\mathbf {\imath }}}\right] \left[ {\mathbf {v}} _{R}\left( t^{\prime }\right) \cdot {\hat{\mathbf {\imath }}}\right] dt^{\prime }\right\rangle ,\nonumber \\ {\dot{D}}_{y}&=-\left\langle \int _{0}^{t_{out}}\left[ {\mathbf {F}}_{f}\left( t^{\prime }\right) \cdot {\hat{{\jmath }}}\right] \left[ {\mathbf {v}} _{R}\left( t^{\prime }\right) \cdot {\hat{{\jmath }}}\right] dt^{\prime }\right\rangle . \end{aligned}$$

In Eq. (12), the term ${\dot{D}}_{x}+{\dot{D}}_{y}$ is the energy dissipated per unit time by the frictional interactions between the slab and the supports*, *whereas ${\dot{U}}_{el}$ is the outflow of elastic energy associated with the residual elastic deformation of the supports leaving the contact, and ${\dot{U}}_{k}$ is the variation per unit time of the supports kinetic energy.

## Results

In this section, we present the main results in terms of the frictional and dynamic behavior of the system. In our calculations, we set $n=$200 and $d_{x}=d_{y}=r_{0}$. Furthermore, we assume the slab size $L_{x}=2L_{y}=$10 $r_{0}$, so that $N_{c,0}=50$. With reference to Eq. (4), friction parameters have been set to $\mu _{s}=1.3$, $\zeta =2.22$, $A=1.44$, $B=-20$, whereas the slab sliding velocity is $V_{0}\approx 10V_{s}$.

In order to explore the frictional behavior of the slab-supports system, in what follows we assume a uniform distribution for $\theta _{0}$ within the interval $[0,2\pi )$. Such an assumption leads to an *in-plane* isotropic response of the elastic support array, which is therefore preferred in applications where the slab sliding direction is not known *a priori*. Results are presented in terms of the normalized apparent friction force $F_{T,x}/\mu _{0}F_{N}$ opposing the macroscopic sliding of the slab in the *x-direction, *where $\mu _{0}=\mu \left( v_{R}=V_{0}\right)$ is the friction coefficient that would be experienced in the case of rigid contact. Moreover, also the apparent friction force occurring in the transverse direction to the slab sliding direction is investigated by means of the normalized quantity $F_{T,y}/\mu _{0}F_{N}$.

**Figure 5:**
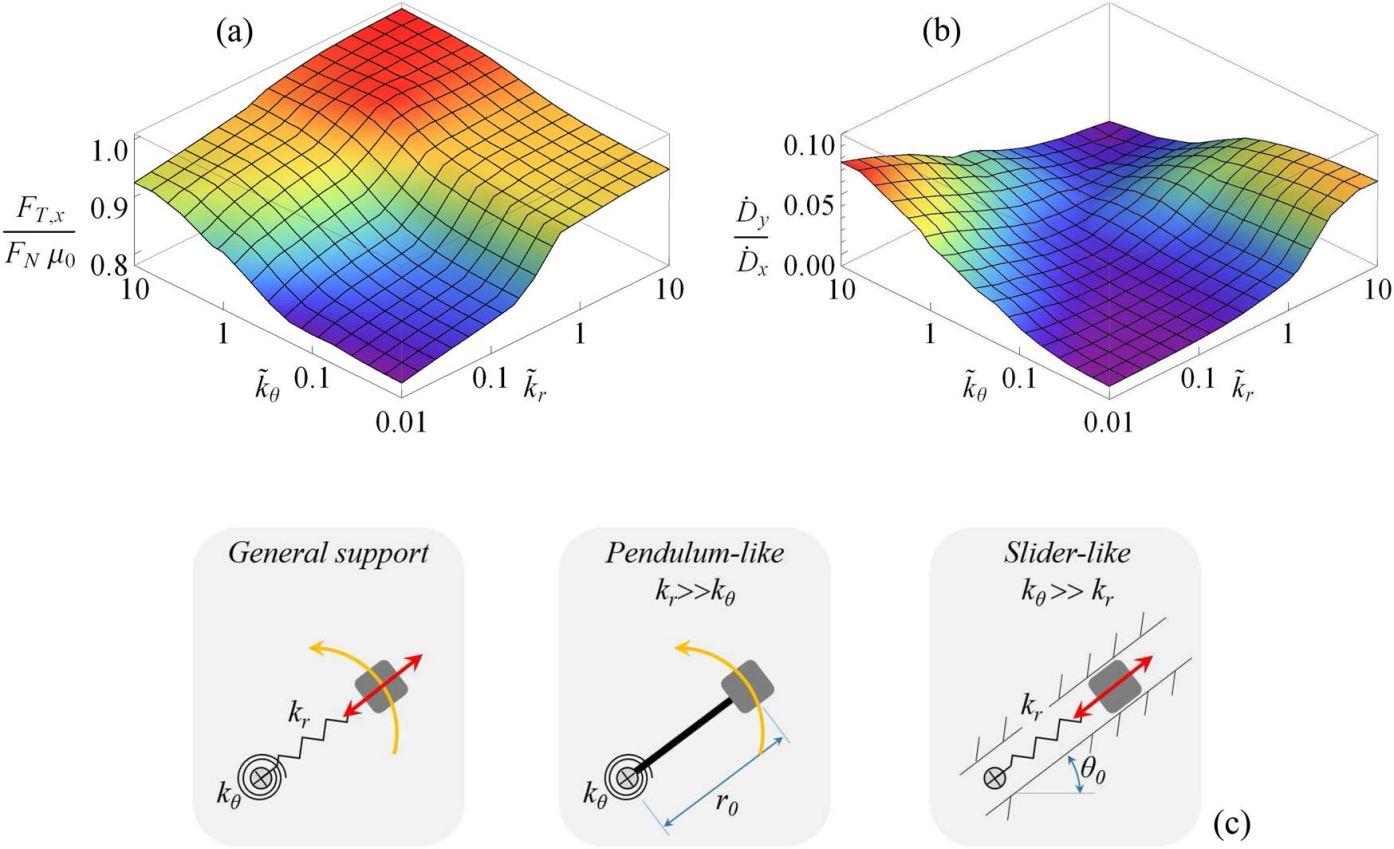
The 3D representations of (**a**) the apparent normalized friction force $F_{T,x}/\mu _{0}F_{N}$ and (**b**) the ratio ${\dot{D}}_{y}/{\dot{D}}_{x}$ as functions of the radial ${\tilde{k}}_{r}$ and torsional ${\tilde{k}}_{\theta }$ stiffness. (**c**) shows a schematic of the qualitative behavior of pendulum and slider dynamic regimes. Results are for ${\tilde{F}}_{N}=1$. Notably $\mu _{0}=\mu \left( V_{0}\right)$.

In Fig. 5a, we show the benefit, in terms of reduction of the normalized friction force $F_{T,x}/\mu _{0}F_{N}$ achievable by interposing elastic supports between the slab and the underlying rigid substrate. We observe that four different regions can be identified, each one associated with a qualitatively different dynamic regime of the supports: the *soft* regime, corresponding to low values of both ${\tilde{k}}_{r}$ and ${\tilde{k}}_{\theta }$, is governed by large elastic deformation of the supports, and low apparent friction is observed; the *pendulum* regime, at large ${\tilde{k}}_{r}$ and low ${\tilde{k}}_{\theta }$, where the dynamics of the supports mainly refers to rigid rotations around the fixed pivot (see Fig. 5c), and a moderate reduction of apparent friction is achieved; the *slider* regime, at low ${\tilde{k}}_{r}$ and large ${\tilde{k}}_{\theta }$, with supports experiencing significant radial deformations with almost constant orientation (see Fig. 5c), again leading to a moderate apparent friction reduction; and the *stiff* regime, with large values for both ${\tilde{k}}_{r}$ and ${\tilde{k}}_{\theta }$, where no significant apparent friction reduction occurs. Figure 5b, instead, shows the ratio ${\dot{D}}_{y}/{\dot{D}}_{x}$ highlighting that both the *soft* and *stiff* regimes are associated with vanishing ${\dot{D}}_{y}$, thus indicating that in these regimes almost unidimensional dynamic behavior along the *x-direction* is experienced*.* On the contrary, both *pendulum* and *slider* regime involve ${\dot{D}}_{y}/{\dot{D}} _{x}\approx 0.1$, which indicates that in these cases the microscopic slab sliding is superimposed to microscopic transverse vibration (i.e. along the *y-direction*) whose effect is to locally modify the relative velocity direction between the supports and the slab, and in turn to reduce the effective friction force opposing the macroscopic slab sliding.

**Figure 6:**
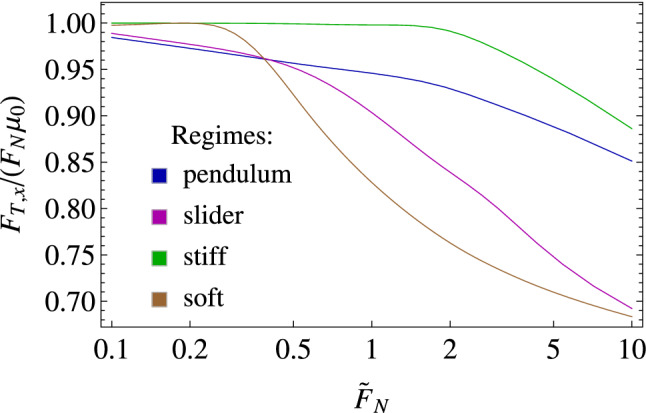
The normalized apparent friction force $F_{T,x}/\mu _{0}F_{N}$ as a function of the dimensionless normal load ${\tilde{F}}_{N}$ acting on the rigid slab. The elastic properties corresponding to the qualitative dynamic regimes are: pendulum, $k_{r}=3$ and $k_{\theta }=0.01$; slider, $k_{r}=0.01$ and $k_{\theta }=3$; stiff, $k_{r}=3$ and $k_{\theta }=3$; soft, $k_{r}=0.03$ and $k_{\theta }=0.03$. Notably $\mu _{0}=\mu \left( V_{0}\right)$.

Figure 6 shows the effect of the dimensionless normal load ${\tilde{F}}_{N}$ acting on the slab on the normalized apparent friction force $F_{T,x}/\mu _{0}F_{N}$ associated with each of the above mentioned dynamic regimes. With this regard, we observe that, since the friction force acting on a single support can be conveniently rewritten as $F_{f}\propto -F_{N}\mu _{s}/N_{c}$, both $F_{N}$ and $\mu _{s}$ have the same effect on the overall system dynamics. Interestingly, Fig. 6 shows that, regardless of the specific supports dynamic regime (i.e. the value of $k_{r}$ and $k_{\theta }$), increasing ${\tilde{F}}_{N}$ leads to a more dramatic reduction of the normalized apparent friction force opposing the slab sliding. Indeed, we observe that the supports are at rest before any interaction with the slab and, once in contact with the slab, interfacial friction is the only source of supports excitation (i.e. ${\mathbf {F}}_{f}$ is the only active term in Eq. (1); therefore, given the values of ${\tilde{k}}_{r}$ and ${\tilde{k}}_{\theta }$, increasing ${\tilde{F}}_{N}$ (as well as $\mu _{s}$) leads to higher frictional interactions and, in turn, to a stronger dynamic response, which eventually exacerbates the mechanics of apparent friction reduction described above.

**Figure 7:**
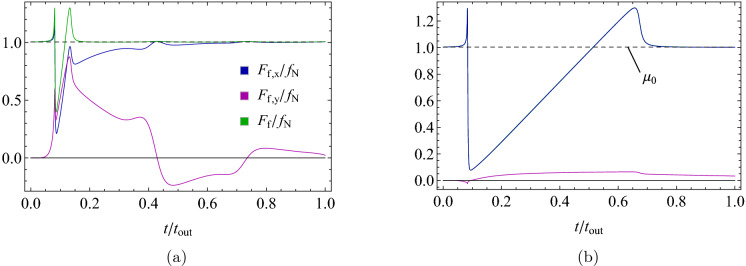
The friction time-history during sliding of a single elastic support with $\theta _{0}=\pi /2$. Results are for (**a**) *pendulum* regime with ${\tilde{k}}_{r}=3$ and ${\tilde{k}}_{\theta }=0.01$ and (**b**) *soft* regime with ${\tilde{k}}_{r}=0.03$ and ${\tilde{k}}_{\theta }=0.03$. Moreover, ${\tilde{F}}_{N}=1.$

Figure 7 refer to a single support dynamics and show the comparison between the normalized friction forces $F_{f,x}/f_{N}$,$F_{f,y}/f_{N}$ and $F_{f}/f_{N}$ during the whole sliding against the rigid slab (i.e. for $t\in \left[ 0,t_{out}\right]$). From these figures, we can better understand the different mechanisms of friction reduction involved in the previously defined dynamic regimes. In particular, Fig. 7a shows the behavior associated with elastic conditions belonging to the *pendulum* regime (notably, similar conclusions can be drawn for the *slider* regime). As suggested by Fig. 5b, the main mechanism here involved in the friction reduction is related to the supports oscillation along the transverse direction (see Fig. 5c) which, indeed, leads to $F_{f,x}<F_{f}$ during part of the sliding process. Interestingly, due to the emerging oscillation in the transverse direction, when $F_{f,x}<F_{f}$ we have that $F_{f,y}\ne 0$. On the contrary, in the case of *soft* elastic supports (see Fig. 7b), we have that $F_{f,x}\approx F_{f}$ (and $F_{f,y}\approx 0$), thus no friction reduction occurs due to transverse oscillations. This time, a different mechanism is responsible for the lower frictional force opposing the slab sliding: since the elastic support is very compliant, a long-lasting “sticky” phase is experienced (i.e. $v_{R}\approx 0$), during which low re-centering elastic force occurs and, in turn, low “static” friction force results. Of course, once the elastic deformation reaches a critical value, the elastic force saturates the “static” friction, and gross sliding occurs with $F_{f,x}/f_{N}=\mu _{0}$. Similar behavior has also been experimentally observed in Refs.^12,16^ for regular arrays of polymeric nanofibers with high aspect-ratio, thus resulting in very compliant behavior. Interestingly, in both cases also $F_{f}/f_{N}>\mu _{0}$ is shortly experienced due to the specific friction law adopted (see Fig. 3).

**Figure 8:**
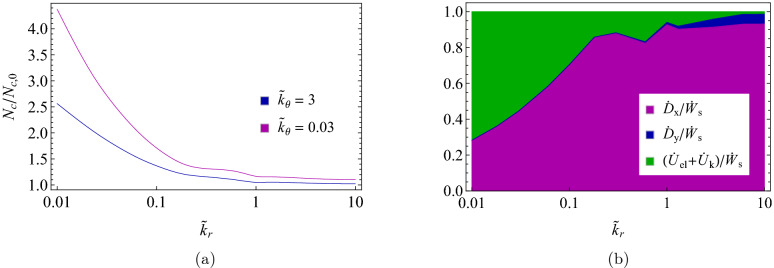
The normalized number $N_{c}/N_{c,0}$ of supports in active contact with slab (**a**), and the breakdown of normalized energetic terms according to Eq. (12) (**b**) as a function of the radial stiffness. Data are for ${\tilde{F}}_{N}=1$, and in (b) ${\tilde{k}}_{\theta }=0.03$.

Figure 8a shows the normalized number $N_{c}/N_{c,0}$ of active elastic supports in effective contact with the sliding slab, where $N_{c,0}=L_{x}L_{y}/d_{x}d_{y}$ is the number of supports underneath the static projection of the slab. Indeed, due to the elastic deformation of the supports caused by the frictional interactions with the slab, we have that $\Delta s\geqslant L_{0}$ and $N_{c}\geqslant N_{c,0}$ (see Fig. 4). Consequently, we observe that, regardless of the value of the tangential stiffness ${\tilde{k}}_{\theta }$, the lower ${\tilde{k}}_{r}$, the larger $N_{c}/N_{c,0}$ is. Interestingly, in the investigated range of radial stiffness, at very low values of ${\tilde{k}}_{r}$, we observe that $N_{c}/N_{c,0}\gtrapprox 2\,$, which means that very elongated supports are still in contact with the slab, thus entailing $x\left( t_{out}\right) /L_{0}\gtrapprox 1$. Of course, the effect of increasing ${\tilde{k}}_{\theta }$ is to globally stiffen the system response, so large values of ${\tilde{k}}_{\theta }$ usually lead to smaller deformations and, in turn, lower values of $N_{c}/N_{c,0}$.

In Fig. 8b, we report the breakdown of the utilization of the work per unit time ${\dot{W}}_{s}$ done by the *in-plane* driving force ${\mathbf {F}}_{T}$ acting on the slab, as indicated in Eq. (12). Indeed, at large ${\tilde{k}}_{r}$ the dynamic conditions belong to the *pendulum* regime, thus most of the energy is dissipated by frictional interactions, as ${\dot{D}}_{x}+{\dot{D}}_{y}\approx {\dot{W}}_{s}$. Moreover, in agreement with Fig. 5, the occurring friction reduction mechanism involves oscillations along the transverse direction, thus the ratio ${\dot{D}}_{y}/{\dot{D}}_{x}$ is nonvanishing. A different scenario is observed for small values of ${\tilde{k}}_{r}$, where a huge reduction of the friction coefficient is reported even when ${\dot{D}}_{y}/{\dot{D}}_{x}\approx 0$. This is the so-called *soft* regime, where most of ${\dot{W}}_{s}$ goes in elastic supports deformation, thus resulting in large $\phi _{el}/{\dot{W}} _{s}\approx 0.7.$

### Effect of supports static orientation

In the previous section, we assumed a uniform distribution of $\theta _{0}$, which is a good approximation to model generic arrays whose static orientation depends on random phenomena such as micropillars buckling or *ad hoc* production procedures^36^. However, since the system dynamics is strongly nonlinear, we expect the specific value of $\theta _{0}$ to significantly affect the behavior of the system.

**Figure 9:**
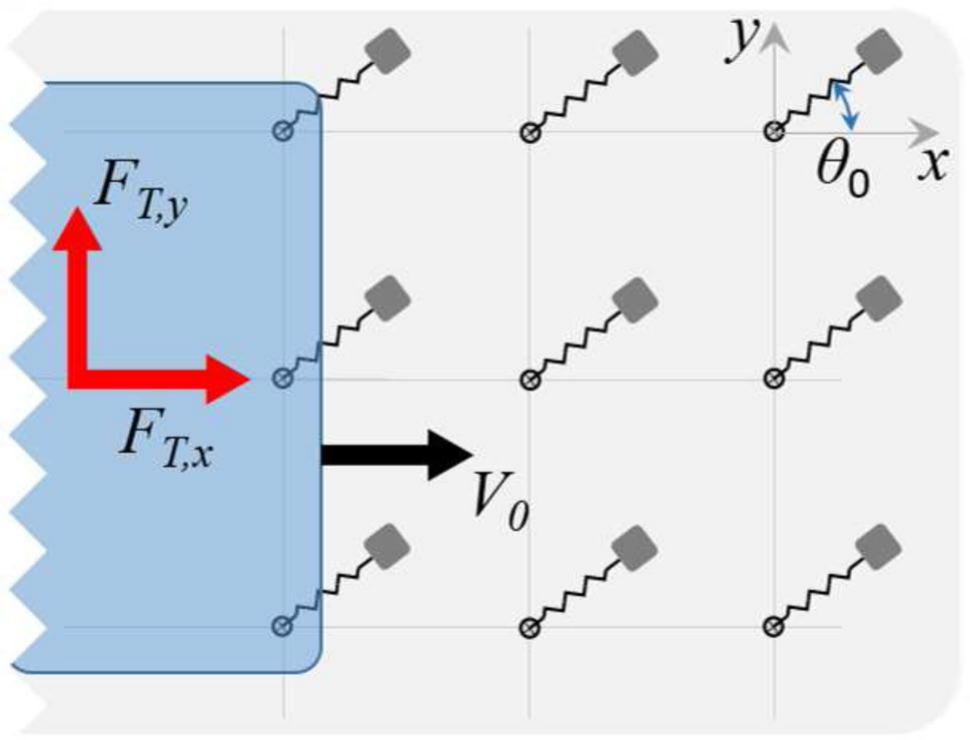
The schematic of the fixed-orientation array case, in which all the supports share the same static orientation $\theta _{0}$. Notably, $F_{T,x}$ and $F_{T,y}$ are the components of the force necessary to keep the slab in steady sliding against the array.

In what follows we focus on the case shown in Fig. 9 of fixed-orientation arrays, i.e. arrays where all the supports have the same static orientation $\theta _{0}$. Notably, in fixed-orientation arrays, all the supports share exactly the same dynamics during sliding against the rigid slab.

**Figure 10:**
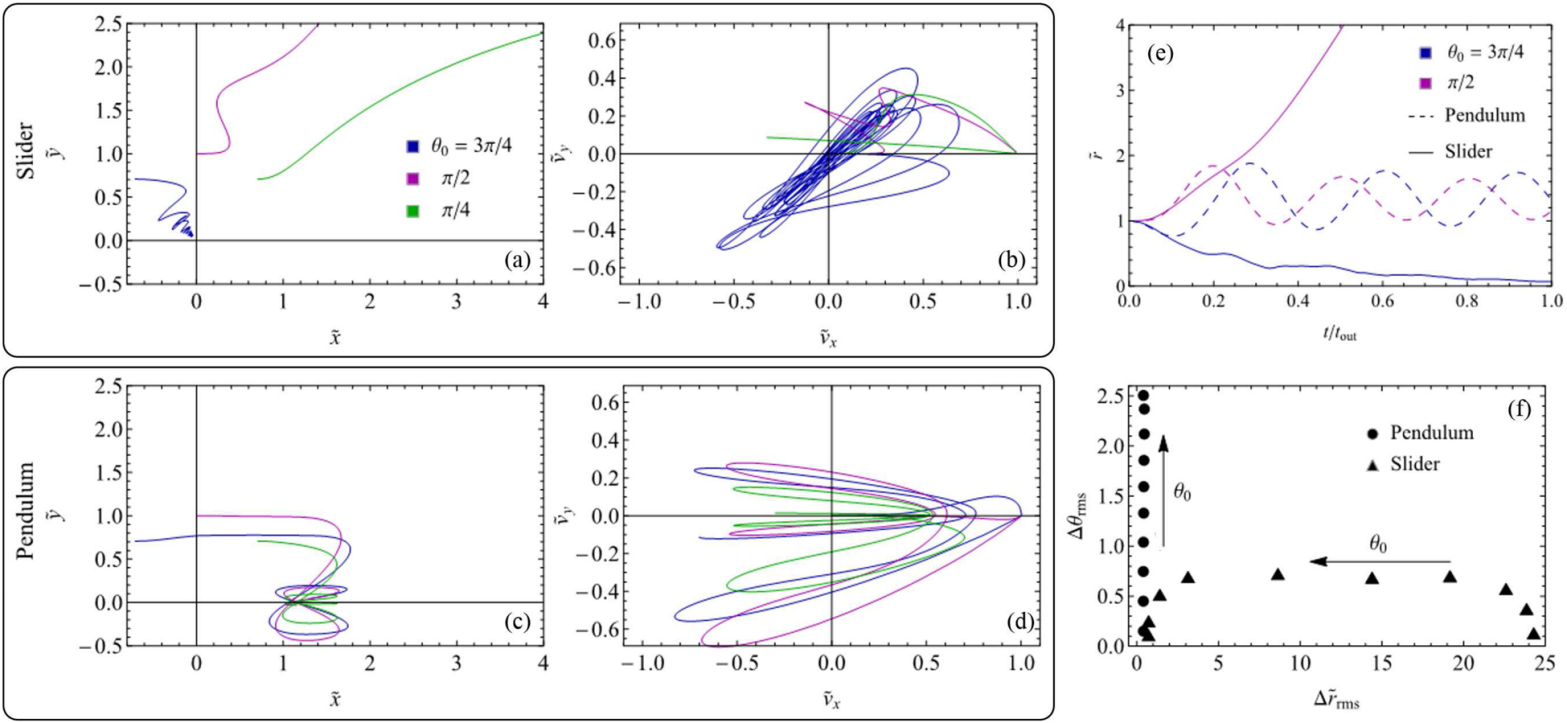
The dynamic behavior of elastic supports extracted from uniform arrays with different static orientation $\theta _{0}$. Displacement (**a**,**c**) and velocity (**b**,**d**) components for supports belonging to, respectively, the *slider *(${\tilde{k}}_{r}=0.01$, ${\tilde{k}}_{\theta }=3$) and *pendulum * (${\tilde{k}}_{r}=3$, ${\tilde{k}}_{\theta }=0.01$) elastic regimes. The time evolution of the dimensionless radial coordinate ${\tilde{r}}$ (**e**) for supports in the previously mentioned elastic regimes with different $\theta _{0}$. The values of $\Delta \theta _{rms}$ and $\Delta {\tilde{r}}_{rms}$ associated with the dynamics of different supports during the whole sliding against the rigid slab under different elastic regimes. Results refer to ${\tilde{F}}_{N}=1$.

The dynamic behavior of fixed-orientation arrays is investigated in Fig. 10. Specifically, in Fig. 10a,b we show, respectively, the displacement and the velocity dimensionless components of three generic supports extracted from three corresponding fixed-orientation arrays presenting different value of $\theta _{0}$ in the *slider* regime. On the contrary, Fig. 10c,d show the same quantities referring to the *pendulum* regime. By comparing Fig. 10a,c we observe that the two regimes under investigation are associated with very different support displacement. Moreover, the *pendulum* regime displacements appear less affected by the specific value of $\theta _{0}$ compared to the *slider* ones. Indeed, in Fig. 10a, due to the stiffer torsional behavior associated with the *slider* regime, peculiarly different behaviors are reported between the case with $\theta _{0}\gg \pi /2$ and those with $\theta _{0}\le \pi /2$, as in the former case, the support dynamics tends to compress the radial spring whereas in the latter condition the radial spring is significantly elongated. Furthermore, Fig. 10b,d allow us to conclude that, in agreement with the discussion provided in the previous sections, in both regimes the maximum values of the *x* and *y* components of the support velocity are of the same order of magnitude, thus indicating that under these conditions the observed reduction in apparent friction can be associated with the effect of transverse vibration. This is peculiarly true for both $\theta _{0}=3\pi /4$ and $\theta _{0}=\pi /2$, whereas for $\theta _{0} =\pi /4$ most of the support motion occurs in the *x-direction*.

Figure 10e,f show the peculiarity of the two dynamic regimes. Indeed, in Fig. 10e we report the time evolution of the dimensionless supports radial coordinate ${\tilde{r}}$ for two different values of $\theta _{0}$ both in the *slider* and *pendulum* regimes. We observe that, regardless of the specific static orientation, in the *pendulum* regime the support elongation oscillates around the value ${\tilde{r}}=1$. On the contrary, in the *slider* regime the value of $\theta _{0}$ plays a key role as for $\theta _{0}\gg \pi /2$ we have that ${\tilde{r}}$ reduces (i.e. the supports shortens), whereas for $\theta _{0}\le \pi /2$ we have that ${\tilde{r}}\gg 1$ (i.e. the supports elongates). Similar considerations can be drawn from Fig. 10f, where we show, for different values of $\theta _{0},$ the values of $\Delta \theta _{rms}$ and $\Delta {\tilde{r}}_{rms}$ corresponding to each support dynamics. These two quantities are defined as the root mean square values of $\Delta \theta \left( t\right) =\theta \left( t\right) -\theta _{0}$ and $\Delta {\tilde{r}}\left( t\right) =\left[ r\left( t\right) -r_{0}\right] /r_{0}$ calculated over the whole support dynamics during the contact with the sliding slab. As expected, the *pendulum* regime is characterized by vanishing values of $\Delta {\tilde{r}}_{rms}$ as, under these conditions, $k_{r}\gg k_{\theta }$. On the other hand, the *slider* regime is associated with $\Delta \theta _{rms}\ll \Delta {\tilde{r}}_{rms}$ as in this case, torsional oscillations are strongly inhibited.

**Figure 11:**
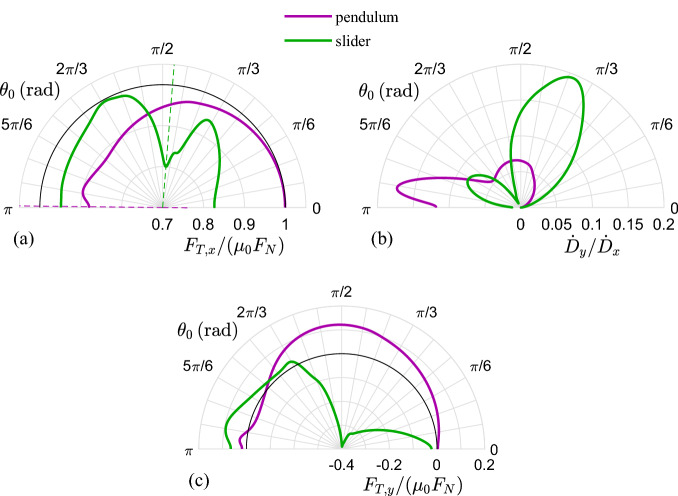
Polar diagrams of (**a**) the normalized apparent friction force $F_{T,x}/\mu _{0}F_{N}$, (**b**) the ratio ${\dot{D}}_{y}/{\dot{D}}_{x}$, (**c**) the normalized apparent friction force $F_{T,y}/\mu _{0}F_{N}$ as functions of the static orientation angle $\theta _{0}$. Two sets of elastic conditions are investigated: *pendulum* refers to ${\tilde{k}}_{r}=3$ and ${\tilde{k}}_{\theta }=0.01$, *slider* is for ${\tilde{k}}_{r}=0.01$ and ${\tilde{k}}_{\theta }=3$. Notably, ${\tilde{F}}_{N}=1$ and $\mu _{0}=\mu \left( V_{0}\right)$.

Figure 11 show the frictional results, in terms of main quantities acting on the rigid slab under steady conditions, associated with fixed-orientation arrays, where all the elastic support have the same static orientation $\theta _{0}$ (see the schematic in Fig. 9). The rationale of the Figures is the following: the quantities (a) $F_{T,x}/\left( \mu _{0}F_{N}\right)$, (b) ${\dot{D}}_{y}/{\dot{D}}_{x}$ and (c) $F_{T,y}/\left( \mu _{0}F_{N}\right)$ are reported as radial coordinates of polar plots, shown as functions of $\theta _{0}$, which is the angular coordinate in the plots. Notably, we only show results for $\theta _{0}>0$ as physical arguments show that $F_{T,x}/\left( \mu _{0}F_{N}\right)$ and ${\dot{D}}_{y}/{\dot{D}} _{x}$ are even functions of $\theta _{0}$, and $F_{T,y}/\left( \mu _{0} F_{N}\right)$ is an odd function of $\theta _{0}$. Again, the results are presented under different elastic conditions (i.e. different values of $k_{r}$ and $k_{\theta }$) belonging to the *slider* and *pendulum* regimes. Specifically, in Fig. 11a the quantity $F_{T,x}/\left( \mu _{0}F_{N}\right)$ is shown, which represents the dimensionless component along the *x-direction* of the external force needed to keep the rigid slab in steady sliding against the fixed-orientation array of supports. We observe that principal orientations can be found, depending on the specific dynamic regime, able to minimize $F_{T,x}$, thus associated with lower apparent friction. Moreover, Fig. 11b, showing the ratio ${\dot{D}}_{y}/{\dot{D}}_{x}$ (see Eq. (13)), allows inferring further arguments on the specific mechanism of apparent friction reduction associated with the considered elastic regimes. Indeed, in both cases, we observe that the ranges of $\theta _{0}$ in Fig. 11a where minimum $F_{T,x}$ occurs correspond to the ranges of $\theta _{0}$ in Fig. 11b where high values of ${\dot{D}}_{y}/{\dot{D}}_{x}$ are reported, thus clearly indicating that in both *slider* and *pendulum* regimes transverse vibrations are mostly responsible for the observed apparent friction reduction. Building on the same path, one can easily argue that fixed-orientation arrays with specific orientations does not present isotropic behavior, thus a non-vanishing component of the resulting force on the slab is expected in the transverse (*y*) direction (see also Ref.^37^). This is investigated in Fig. 11c, where the normalized apparent friction force $F_{T,y}/\left( \mu _{0}F_{N}\right)$ is shown as a function of the array static orientation $\theta _{0}$. As expected, for $\theta _{0}\rightarrow 0,\pi$ a vanishing value of $F_{T,y}$ is reported. Interestingly, we observe that, in the *pendulum* regime, $F_{T,y}\ge 0$ regardless of the value of $\theta _{0}$ as the elastic supports are always elongated (see Fig. 10e); whereas, in the slider regime, the value of $\theta _{0}$ also affects the sign of $F_{T,y}$, as depending on $\theta _{0}$ the supports can be elongated or shortened during the contact with the slab.

From these results, it follows that the frictional behavior of a generic array could be interestingly tuned by arranging the static orientation $\theta _{0}$ of its supports to a proper distribution. In this view, also the resulting transverse friction force $F_{T,y}$ can be controlled by balancing the supports with orientation $\theta _{0}$ with those with orientation $-\theta _{0}$, thus leading to* in-plane* orthotropic array behavior.

## Conclusions

In this work, we investigate the dynamic and frictional behavior of a regular array of *in-plane* elastic supports interposed between a sliding body and the underneath substrate. We show that, by introducing the elastic supports, a significant reduction of the overall friction force opposing the macroscopic sliding of the sustained body can be achieved compared to the case of rigid contact with the substrate. Indeed, depending on the specific *in-plane* elasticity, different dynamic regimes of the supports can be observed, each of which associated with a specific support frictional behavior. The friction reduction mainly occurs via two alternative mechanisms: for stiff supports, local microscopic transverse oscillation of the supports occurs, which deviates the effective friction force direction from that of the macroscopic sliding; for compliant supports, the poor elastic reaction force of the support lead to long-lasting local “static” friction conditions at the interface.

Interestingly, the supports static orientation plays a key role in the final dynamic behavior of the array, and in turn on the frictional response of the foundation. When the macroscopic sliding direction of the sustained body is not known *a priori,* random uniform distribution of the static orientations can be adopted to ensure *in-plane* isotropy; however, practical applications involving known sliding directions can benefit from a deterministic design of the supports orientation able to further reduce the frictional force acting on the sliding body.
